# A Real-Time Streaming System for Customized Network Traffic Capture †

**DOI:** 10.3390/s23146467

**Published:** 2023-07-17

**Authors:** Adrian-Tiberiu Costin, Daniel Zinca, Virgil Dobrota

**Affiliations:** Communications Department, Technical University of Cluj-Napoca, 400114 Cluj-Napoca, Romania; costin.t.adrian@gmail.com (A.-T.C.); virgil.dobrota@com.utcluj.ro (V.D.)

**Keywords:** network traffic logger, Libtins, Apache Kafka, ksqlDB

## Abstract

Logging network traffic offers valuable insights into data flow, enabling the proactive analysis and troubleshooting of issues as they arise. Moreover, it provides a means to access and examine the exchanged information among network users that would otherwise be inaccessible. To enhance network traffic analysis, the integration of innovative technologies that facilitate real-time querying and pattern matching proves indispensable. This research paper presents a system that exemplifies such advancements—an innovative network traffic logging tool. The tool specifically focuses on performing real-time network packet transfer to Apache Kafka and ksqlDB, leveraging their capabilities to ensure swift and dependable storage of network packets in Apache Kafka topics. By showcasing this solution, the paper demonstrates the benefits and effectiveness of employing modern technologies for network traffic analysis and management.

## 1. Introduction

Logging network packets can prove vital for monitoring the ever-changing flow of data inside a system, as it allows the engineer to quickly spot problems, troubleshoot and perhaps fix them before they become major issues.

In today’s interconnected world, computer networks play a vital role in facilitating information exchange. As network usage grows exponentially, understanding and managing network traffic becomes increasingly crucial.

One valuable tool in this regard is traffic logging, which can be described as capturing and analyzing network data for various purposes. It involves capturing packets of data as they traverse through a network, extracting relevant information, and storing it in log files or databases for further analysis. These packets provide valuable insights into network activity, including the type and volume of traffic, source and destination IP addresses, protocols used, and much more information on Layers from 2 to 7.

Some of the benefits of traffic logging and analysis include troubleshooting and optimization of network performance, intrusion detection, capacity planning, and resource allocation. By analyzing network traffic, the network administrator can identify which applications or devices are consuming excessive bandwidth, and this information is the first step in the process of optimizing network resources and prioritizing critical traffic. Furthermore, when monitoring network traffic patterns, anomalies, unusual behavior, and other categories of intrusion detection can be detected, enabling prompt response and mitigation. Additionally, network traffic analysis can also provide valuable insights for capacity planning by monitoring traffic trends and forecasting future growth.

To be effective, the process of saving the capture must happen in real time. Adding to that point, due to the complexity and diversity of network traffic packets and considering confidentiality, the fields saved by the traffic logger must be customized. No network logger should save the message payload because it can contain user-confidential data. Moreover, depending on the application, certain header fields on each layer are needed.

All traffic should be captured, including malicious packets. To capture real-time traffic, processing should be kept at a minimum. Any additional data operations, such as traffic classification into normal or malicious categories, should be conducted separately as a distinct process that utilizes the captured data. Special measures must be taken to prevent attackers from gaining control over the network logger system to ensure that the logging process is not tampered with and that the information that is saved accurately represents the packets generated in the network.

A customized network logger should enable independent storage of individual fields extracted from each protocol header. Due to the immense volume of packets transferred on high-speed networks, it is necessary to store log information in streaming systems rather than traditional files, as the latter would become excessively large. Further processing of large files can be challenging and inefficient due to their size and complexity. Using streaming systems for storing network logs offers real-time processing, scalability, low latency, flexibility, and seamless integration with analytical tools, enabling efficient and effective analysis of network activities.

These benefits can be further enhanced by utilizing a tool such as Apache Kafka, “an open-source distributed event streaming platform used by thousands of companies for high-performance data pipelines, streaming analytics, data integration, and mission-critical applications.” [1] and ksqlDB, a database “purpose-built for stream processing applications” [2]. Storing the network data in these can prove quite useful as they allow for real-time pattern matching using queries, as seen in [3].

This paper partly aims to showcase how such a network logging tool can be implemented using the Libtins library [3], with the main provided functionalities being the ability to intercept network packets from a live interface or read them from a capture file, the ability to process the captured packets in a way that introduces changes to them, and the ability to forward those packets to a specific destination in real time.

### 1.1. Libtins

Libtins is a “powerful C++ network packet sniffing and crafting library” [3] that provides developers with extensive capabilities for network traffic analysis and manipulation of multiple protocols. The packet capture is based on the Libpcap [4] library. At its core, Libtins offers packet sniffing functionality, allowing developers to capture network packets from different network adapters (that run under various operating systems) and analyze their contents. It supports a wide range of protocols and encapsulation schemes (including but not limited to Ethernet, IP, UDP/TCP, and HTTP), enabling efficient parsing and inspection of network protocols. With Libtins, header fields and payload data can be easily extracted from captured packets, facilitating deep packet analysis and protocol-specific processing. In addition to packet sniffing, Libtins enables packet crafting and injection, empowering developers to generate custom network packets for testing, simulation, or security-related tasks. The packet injection capabilities of Libtins enable developers to inject crafted packets into the network, facilitating testing, intrusion detection, or other network-related experiments.

A more common alternative to Libtins is Libpcap [4], a C library focused primarily on packet sniffing. It provides a lower-level interface, mainly focusing on packet capture and filtering capabilities. Libpcap captures packets at a lower level, providing raw packet data. It does not include built-in parsers for specific protocols, requiring developers to implement their own parsing logic to extract protocol-specific information. Libpcap focuses primarily on packet capture and does not include built-in functionality for packet crafting or injection. As stated in [3], Libtins exhibits one of the lowest processing delays compared to other libraries, ranking second only to Libpcap. To build a customized packet capture solution, Libpcap requires extensive programming to define specific fields for each analyzed protocol. 

Libtins provides a higher-level API with support for protocol parsing, packet inspection, crafting, and injection, making it suitable for more advanced network packet analysis and manipulation tasks.

The power of the Libtins library comes from the fact that in numerous ways it acts as a wrapper over the lower level Libpcap, granting the user the reliability and speed provided by this library in the context of network packet capture, all packaged in an easy-to-use interface. Relying on these abstractions that make Libpcap calls under the hood makes it intuitive and easy to use, without sacrificing performance.

To ensure fast and reliable storage of network packets, it wraps the raw data into high-level objects called Protocol Data Units (PDUs). These are then layered on top of each other to create a full protocol stack, from the Data Link layer up to the Application Layer. All these operations are performed seamlessly immediately after the packet is captured.

In the context of our research paper, specifically focusing on network traffic capture and logging, we opted for this library due to its remarkable utility. With minimal limitations for the use-case we proposed, and significantly simplifying our tasks compared to directly relying on the lower-level Libpcap, it was an evident and advantageous choice.

### 1.2. Apache Kafka

Apache Kafka [1] is a distributed streaming platform that enables the processing, storage, and analysis of real-time data streams at scale. It was designed to handle high-throughput, fault-tolerant data streams and has gained significant popularity due to its robust architecture and versatile features.

At its core, Kafka operates as a distributed publish-subscribe messaging system. It introduces the concept of topics, which represent streams of records or messages. These topics are divided into partitions, allowing for parallel processing and increased throughput. Producers are responsible for publishing records to Kafka topics, while consumers subscribe to these topics and process the incoming messages. Kafka’s distributed architecture ensures scalability and fault tolerance. It uses a cluster of servers, called brokers, to store and manage the data. Each broker can handle multiple partitions, providing horizontal scalability as the cluster size increases. Additionally, Kafka replicates partitions across multiple brokers, ensuring data availability and durability even despite failure.

One of Kafka’s key features is its ability to support real-time stream processing. It allows developers to build applications that can process and analyze data as it flows through the system, enabling low-latency data processing and real-time insights. Kafka’s stream processing capabilities have been enhanced through the integration of Apache Kafka Streams: a lightweight stream processing library built on top of Kafka.

Kafka offers several benefits for data processing architectures. It acts as a central nervous system for a modern data infrastructure, enabling the integration of various data systems and applications. Kafka also supports connectors that facilitate the integration of external data sources and sinks, making it easier to ingest and export data. Apache Kafka has been widely adopted across industries and has proven to be a valuable tool for various use cases.

Due to the requirements of applications that process network packets in real-time, such as Intrusion Detection Systems and Flow Analysis tools to name a few, we can argue that using a real-time data streaming platform such as Apache Kafka provides numerous advantages.

First, the ingested packets are neatly arranged into topics inside the database. This translates to good data management. Furthermore, the provided storage is fast access, greatly improving the speed of applications using it as a backend.

Furthermore, due to the real-time capabilities of this technology, the ingested data can be processed immediately, resulting in remarkable response times with minimal delays. In our case, this means that the network packets ingested into Kafka can be queried immediately and the system can determine if they fall into a specific category sooner rather than later. This means that for an Intrusion Detection System, for example, a query searching for malicious activity could detect intruders trying to breach the system quicker than if we were to rely on other more dated methods.

Another major advantage of using this as opposed to other technologies is that, because of its nature, Kafka is a distributed data storage system, where producers push data to the database all the time, sometimes in parallel, whilst consumers read the data and act based on it. This means that it greatly eases the scaling of software projects that use it. In our case, this would mean granting the ability to monitor multiple sites at the same time, with easy-to-use queries that can provide a clear picture of the state of those specific networks, from afar.

### 1.3. ksqlDB

ksqlDB [2] is an open-source streaming SQL engine built on top of Apache Kafka that enables developers and data engineers to process and analyze real-time streaming data using SQL-like syntax. It provides a high-level, declarative interface for working with streaming data. ksqlDB extends the capabilities of Apache Kafka by introducing SQL-like queries and transformations for streaming data.

With ksqlDB, users can define streams and tables as first-class abstractions over Kafka topics, allowing them to treat streams of events as continuous, structured data sources. This stream-centric approach simplifies the development and maintenance of real-time applications that require continuous processing of data.

The Windowing and Time-Based Operations feature of ksqlDB provides support for windowing operations, enabling users to perform aggregations and calculations on data within specific time intervals. This capability is crucial for analyzing time-sensitive data and deriving insights based on time-based patterns.

Continuous Queries is the ksqlDB feature which allows users to define queries that frequently process, and update results as new data arrive. This real-time processing capability is essential for monitoring, alerting, and generating live insights from streaming data.

ksqlDB helps in building applications targeting various real-time data processing scenarios, including real-time analytics and dashboards, because it allows users to generate real-time insights and build interactive dashboards by analyzing streaming data as it arrives.

By continuously processing and analyzing data streams, ksqlDB can identify patterns and anomalies in real-time, enabling Anomaly Detection Systems. Also, rule-based Intrusion Detection Systems can be implemented by using queries [5].

### 1.4. Existing Work

Traffic capture involves the collection and analysis of data packets to gain a deeper understanding of network behavior, identify security threats, and optimize performance. It provides valuable insights into the inner workings of the infrastructure.

By analyzing captured packets in a specific network, administrators can understand traffic patterns, identify bottlenecks, and determine the root causes of performance issues. These insights help in making informed decisions regarding optimization, capacity planning, and resource allocation.

One of the most common tools is Wireshark [6], which can use Libpcap or other related libraries to capture live network traffic and save it to a file with a pcap (or similar) format. In many situations, developers may use libraries like Libtins or Libpcap to build specialized applications that focus on certain aspects of network traffic. For network traffic logging applications that store relevant information, a corresponding delay is unavoidable and of significant importance. 

Among other streaming technologies, Apache Kafka was used by several research papers to provide storage for network traffic:

1. In [7], the authors propose a mechanism that extracts information from the network using the Cisco Netflow protocol and then uses Kafka topics to implement real-time anomaly detection using three Machine Learning algorithms.
2. Kafka is also used in [8] as an event log database that collects log messages generated by network and software processes.
3. Apache Flume can be used for the process of log collection and ingestion into Kafka as described in [9].
4. In [10], the reliability of high-volume data stream ingestion into Kafka was investigated using a custom-developed tool.
5. Ref. [11] uses Kafka and Apache Spark Streaming to detect cybersecurity attacks, and based on the UNSWNB-15 dataset, it was concluded that this architecture has a good performance in terms of fault-tolerance and processing time when presented with huge amounts of network traffic.
6. A Kafka streams-based classification of DDoS attacks presented in [12] uses the CICFlowMeter tool [13] to extract features from the network flows. The CICFlowMeter tool extracts 83 statistical network traffic features and saves them on a csv file. These features are appropriate for Machine Learning Intrusion Detection Systems. From the csv file containing more than 80 features for each record, ref. [12] describes how a Kafka connect agent publishes network flows onto a streaming-flows topic.
7. In our previous papers [14,15], we had a similar sequential approach by separating the capture process from the ingestion into Kafka topics. The paper is an extended version of reference [16] that was presented at the International Symposium on Electronics and Telecommunications and proposes a multi-threaded architecture where the packet capture and the ingestion processes run in parallel, offering a much better performance and paving the way for future improvement of existing applications.

In the following sections, a system for customized network traffic capture is described. Starting with 1.5, the central element of the proposed system is introduced, namely the “Netlog v1.0.0” application we developed, and integrated into a more elaborate system where it communicates with Apache Kafka and ksqlDB. Onwards, in Section 2, we attempt a deeper dive into the inner workings of the proposed solution, focusing on showcasing the advantages and the mechanism of deploying it in practice. The complete source code of Netlog is provided in [17].

### 1.5. The Proposed System for Network Traffic Capture

**Figure 1:**
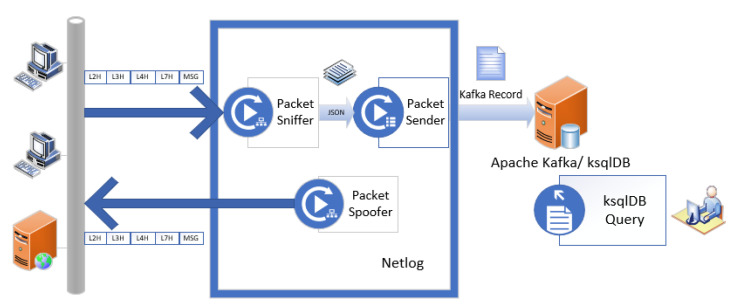
Block diagram of the proposed system for network traffic capture.

Figure 1 presents the proposed system for network traffic capture. We developed the Netlog component that captures network traffic, processes it, and inserts custom fields from each packet into an Apache Kafka topic. A network administrator can run ksqlDB queries against topics in the Kafka database as illustrated in [5,14], and this falls outside the scope of this paper’s objectives.

The overall architecture of the Netlog v1.0.0 application is presented in Section 2.1. The Netlog component consists of three main modules. The network traffic generated by the computers and servers on the monitored network is captured in real-time using the Packet Sniffer module, as will be described in Section 2.3. Header information from the L2, L3, L4, and L7 layers is extracted using the Libtins library. That information is then converted to JSON format, as will be described in Section 2.4, before being sent for ingestion in an Apache Kafka topic using the Packet Sender module. Depending on the protocols used in the capture process, generating packets may be useful. For this task, the Packet Spoofer module was developed and is described in Section 2.5.

### 1.6. Netlog: The Network Traffic Logger

This section introduces Netlog [17], the application we developed with the purpose of improving the performance of ingesting network traffic captured with the aid of the Libtins library into Kafka. From a top-level view, to provide the sniffing functionality, our application uses an abstraction that wraps the packet capture API provided by the Libtins library. Building on top of that, an extensible design is used to enable spoofing and forwarding the packets. This, in turn, is highly extensible and allows users to define their own algorithms according to a specific use case. Whether it is just logging the traffic locally, or perhaps forwarding it to another network endpoint, or storing it inside a database for future queries and processing, our approach provides a functional layer acting as a base for a more elaborate software stack.

Furthermore, by employing the aforementioned method, developers can use a similar tool that offers a high degree of customization regarding the intended processing applied to the captured packets and the rules for forwarding data. This process is showcased with some already defined examples, but the user can also roll their own custom algorithm with a bit of extra source code, in an intuitive fashion. All of this is made possible through a simple to follow, yet powerful design.

Keeping that in mind, during the following sections, we shall discuss some of the details that go into developing a packet capture tool that has the capability of interacting with modern technologies such as Apache Kafka and ksqlDB in real time. Moreover, to see how it all bonds together, we will analyze the architecture of such a tool, emphasizing the design choices that make all the things described above possible.

## 2. Materials and Methods

The main flow of the application can be broken down into three parts: the sniffing process, the packet spoofing interface and the packet sending interface. The user can select between these branches of execution by providing different arguments to the command-line interface.

Due to its multi-threaded design, our application ensures that processing the captured network packets and forwarding them to the Kafka topic happens in parallel. This, in turn, greatly improves the performance of the application when compared to the sequential approach used during the development of [14], which required separating packet capture and Kafka ingestion.

In the following subsections, we will walk through how this functionality is implemented, first taking a bird’s-eye view and later diving into each component in more detail to better explain the design choices and showcase their importance in making it all work.

### 2.1. Overall Architecture of the Netlog Application

Taking a top-level view, we can break down the application into several modules. The main component that dictates the flow of the software project is the Application module. This class is responsible for ensuring proper communication between all the other software components. It communicates with the command-line argument parser, the packet capture, packet spoofing and packet forwarding modules, respectively.

The application uses a custom command-line argument parser, specifically designed for handling all the user-specified parameters via the command-line interface. This module reads and communicates data ranging from capture filters, interfaces, and sniffer types to broker and topic information relevant to the Apache Kafka data transfer.

The execution flow demands that the Application module spawns an instance of the second most important module, called PacketCapture. This one is responsible for spawning a Libtins Sniffer that will use Libpcap to capture all the network traffic from the specified interface, or from a packet capture file.

Afterwards, the application communicates with the PacketSender module, which ensures swift data delivery to one of the specified endpoints, which in our use-case is Apache Kafka, whilst also notifying the user with a message delivery report.

### 2.2. Creating the Application

The main entry point for the network traffic logger is the Application class, which is an abstraction that helps organize and manage all the objects present in the program, handling their interactions to sniff network traffic, spoof it, and forward it.

**Figure 2:**
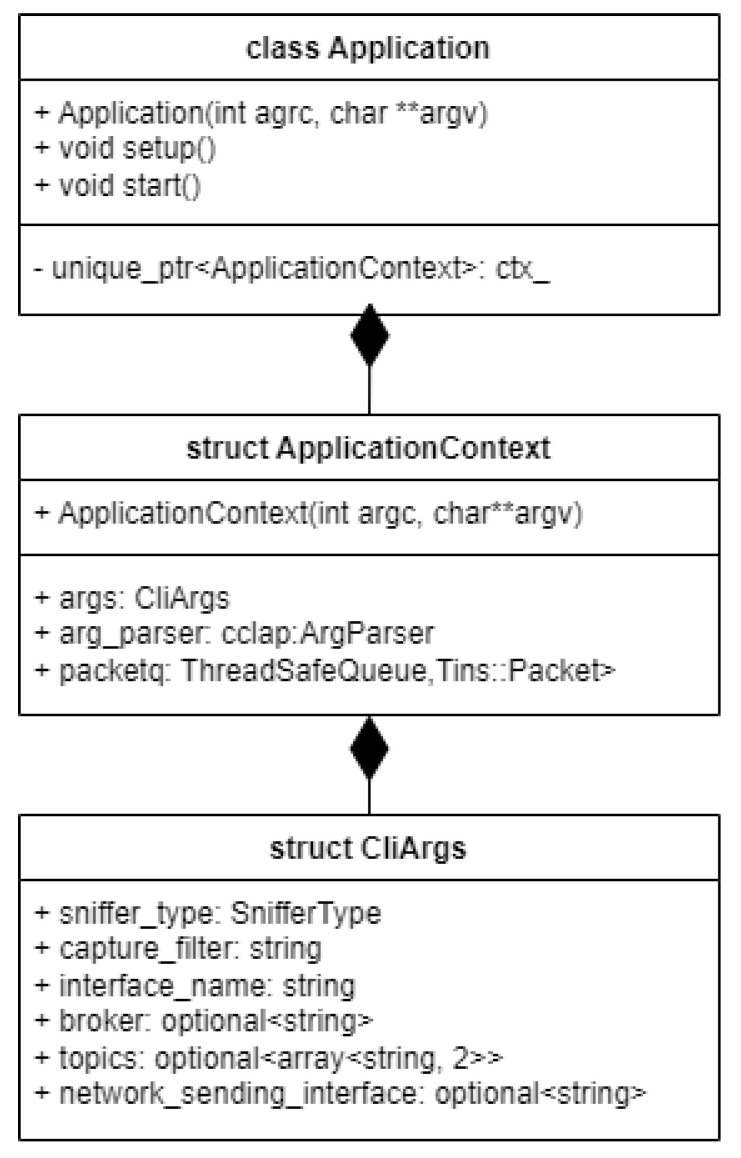
Application Class.

As presented in Figure 2, the inner objects of the application are all stored in a context, which holds the CLI arguments, the sniffer, sender, spoofer, and an intelligent queue, designed to act as intermediary storage for the captured network packets whilst being thread safe. The packets from this queue are then popped one by one, converted to JSON, and sent to the destination in real-time. An intermediary spoofing process can also be run if the user wants to modify the captured packets before sending them to the destination.

To tie all the components together and make the application interactive, a custom command-line interface argument parser is used, which processes all the input the user gives to the program and stores it inside an appropriately named data structure.

To generate an application object, the command line arguments are forwarded to the Application class, which uses them to create the ApplicationContext, where these arguments are consumed by the command line argument parser. Afterwards, the setup method is called to process the arguments and store them in the CliArgs data structure.

The main top-level logic that gets executed is implemented inside the “start” method. This consists of spawning two execution threads, out of which one will handle capturing the network packets and storing them into a queue, effectively acting as a producer, whilst the other will consume packets from the queue if they are available and process them, followed by executing a sending operation to the desired endpoint. If a regular sniffer is used and the user chooses to capture traffic on a live network interface, the application also spawns a third user interaction thread, which does the key event polling work to ensure the packet capture can be stopped on keypress.

### 2.3. Sniffing Network Packets

**Figure 3:**
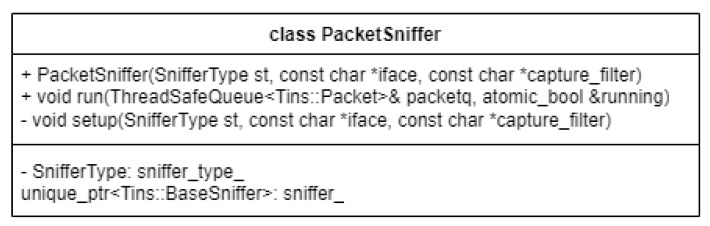
Packet Sniffer.

The packet capture process is handled in one of two ways. The options the user is given are distinguished using command line arguments. The first one is to sniff the packets from a live network interface, whilst the second involves reading them from an input packet capture file. In Figure 3, we can see the API provided by our PacketSniffer class for capturing network packets.

The distinction between these two use-cases is made automatically, depending on what the user passes as a command line argument for the input interface. This parameter, intuitively named “interface”, controls the type of packet sniffer that will be created by the application. Depending on this input, the application will choose to construct either a standard Libtins Sniffer or a FileSniffer. This way, the application targets both types of network packet sniffing based on the requirements of the user.

If an existing network interface is specified, along with the “live” capture option, the program selects that network interface or uses the default one if it is invalid. On the other hand, if the live capture is not requested, the application assumes that whatever input the user provides is a file name from where network packets can be read.

The underlying mechanism of this abstraction is straightforward. Using basic principles of Object-Oriented Programming, such as inheritance and polymorphism, the PacketSniffer constructor creates a custom sniffer based on the presented command-line arguments, and afterwards references it through a C++ smart pointer of the base type inside the created object. All this functionality is abstracted away inside of the private setup function we can see in Figure 3. The type of sniffer that was constructed is also stored, for later use.

In addition to the abstraction of the input type using command-line arguments through the constructor for this custom PacketSniffer type, as mentioned above, we can also observe that a “run” method is also exported through the freshly created sniffer object, making different types of packet capture or reading operations as easy as calling this function once we instantiated the previously mentioned constructs. The way this works, in the context of the Libtins library, is through callback functions.

Our PacketSniffer “run” method directly starts a “sniffing loop”, which is a Libtins concept for starting a continuous packet capture that does not stop until all available packets are consumed. For a “.pcap” file, this would be when the end of the file is reached, while for a live network interface this would mean an infinite loop, at least until the user manually breaks out of it or the network connection suddenly stops working. To combat this, our application provides a “stop on keypress” feature, which uses the “running” boolean variable that can be seen above to halt the sniffing when the user presses a designated key.

To further elaborate on the Libtins callback functionality mentioned above, we can summarize it as an action that is taken for each captured packet. Inside the library source code, the creator provided his users with the option to bind a callback function to the “sniff loop”. This callback function will then be executed every time a packet is intercepted in the sniff loop. Inside of the callback function, the user can then do packet processing and split the captured packet into its individual layers, called PDUs. This is further discussed in the Libtins documentation.

In our case, the action we take when a new packet is intercepted is to simply store it into a ThreadSafeQueue type, which is a custom construct that will help us parallelize our packet storage in memory, to be able to access it from different threads of execution. This is important because by going this route, we pave the way to faster execution and packet processing speed, ensuring that our captured packets are processed at pretty much the same rate as they are captured; thus, we are not limited by the packet processing time in the same way as we would be if we decided to process them sequentially. This approach provides us with far better results than the previous one detailed in [14], where all the captured packets were stored in memory using a C++ vector datatype and later processed individually and in sequence after the capture managed to consume all the desired packets, thus making the packet processing at least linear in time complexity, before considering the actual algorithm applied to each packet.

One more important feature of the PacketSniffer objects is the fact that they allow the user to provide a custom packet capture filter. These filters have to be provided in a specific format and used by Libtins and forwarded to the lower level Libpcap API. This lower-level library knows how to process the capture filter and provides the user with only the desired packets based on it. Providing a capture filter of “port 5060”, for example, as in [1] would allow the user to intercept only VoIP packets as this port is used for sending and receiving SIP packets, which are used in the call initiation process in IP telephony. This functionality is not actively used in our examples but is present if the other use cases demand it.

### 2.4. Packet Conversion to JSON Format

Converting Libtins packets into an intermediate format is important as communication with other systems is greatly eased by using a common format such as JSON.

This translation is performed using the rapidjson library, a “fast JSON parser/generator for C++ with both SAX/DOM style API” [15]. The main reason behind choosing it is that it is “small but complete”, fast, self-contained, and memory friendly.

The JsonBuilder type is essentially a “view” into the captured network packet, which allows the user to access the internal state of the stored packet through some pointers defined in the PacketAdapter private member data type. The main reason for its existence is to allow the user to create JSON strings based on the stored packets, and our application mainly uses this to convert the data to this format before executing a sending operation, as this ensures communication using a common format between different application layers or tools. This makes integration in different workflows easier and is a huge benefit for our specific use-case, in which we want to communicate with Apache Kafka, which uses this specific format in some cases for data exchange.

A public constructor is exposed, which provides a way for the user to instantiate an object of this type by providing a Libtins network packet and a JSON writer object from the RapidJSON library. The former is provided from memory, if a network packet is available, and the latter is created dynamically every time we construct an object of this type.

To create a JSON string, a public method called “build_json” is provided, which uses the internal functionality of the builder to append each network layer to the output string for the specified packet.

The process by which the captured network packet is converted to JSON can be detailed in the following way. Firstly, a network packet adapter is used, containing pointers for each of the protocols that can be processed by Libtins (datalink, network, transport, and application layers). Each of these are retrieved by using the Libtins API and stored in memory. The access to these resources is carried out through the previously mentioned pointers. After processing and storage in memory, the network packets are filtered and grouped based on the protocols they are made up of to ensure that the data can be successfully converted into JSON.

**Figure 4:**
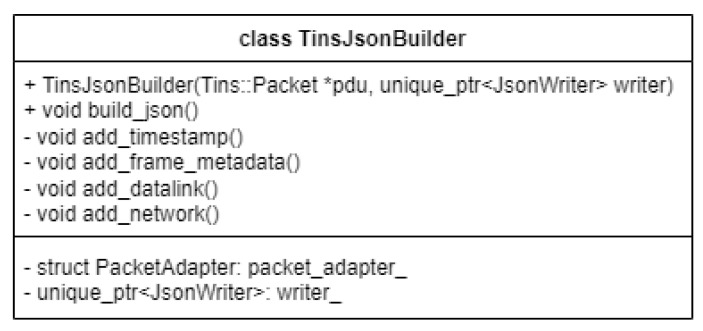
JSON builder class.

After organizing and determining the PDUs that make up the network packet, the implemented JsonBuilder class selects the present protocols and starts building a JSON output string from the ground up using functions from the RapidJSON API. The structure of this custom type used for building JSON strings can be seen in Figure 4.

### 2.5. Spoofing Network Packets

To spoof network packets, an extensible interface is provided, which allows the user to pick one of the available algorithms that operate on libtins network packets, or even define their own by extending the SpoofingStrategy class.

**Figure 5:**
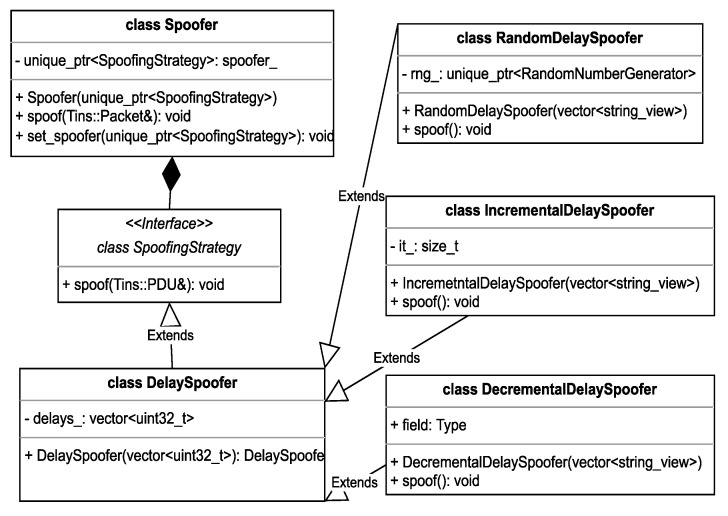
Packet Spoofer.

As can be seen in Figure 5, the SpoofingStrategy interface is extended by multiple types of spoofers. A few example spoofers have been provided like the RandomDelaySpoofer, IncrementalDelaySpoofer, and DecrementalDelaySpoofer.

The intention behind the examples mentioned above is to showcase the process a user can follow to create his own custom spoofers. Extending the functionality is simple, as the user only must provide an extra class which implements the spoof method and adds the desired functionality for addressing the custom target use-case.

The readily available examples rely on modifying the timestamp field of the Libtins Packet object by adding a variable amount of delay to it, using different patterns. This is carried out by relying on an input array of delays that the user provides, from which values will be picked according to the type of spoofer, either randomly or using an ascending or descending type of element indexing.

To integrate all components and to ensure seamless operation, a spoofing context is used, in the form of the Spoofer class. This class holds the chosen spoofer as a member object and uses its implementation of the spoof method to modify the input network packet, whilst also providing an option to switch between the configured spoofers using the “set_spoofer” method.

### 2.6. Real-Time Network Packet Sender

**Figure 6:**
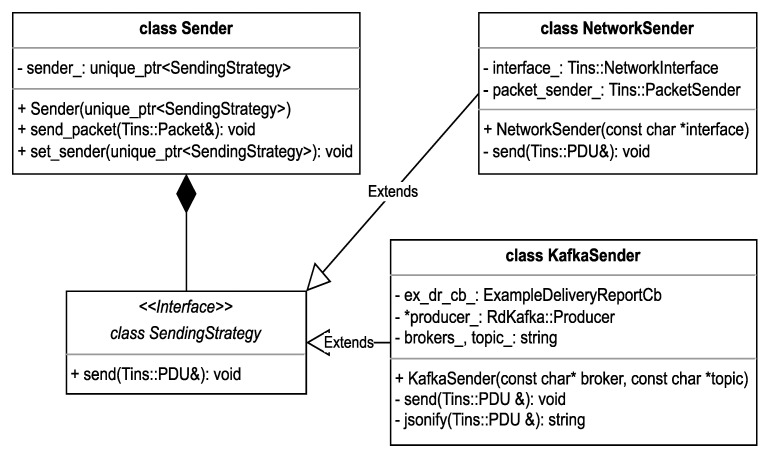
Packet Sender.

To send the captured packets in real-time to the specified destination, a network packet sender is used. Much like the spoofer, we use the same strategy to differentiate between the different types of senders that are available. This can be seen in Figure 6, where we provide a more detailed overview of the Sender design, which involves an extensible interface for configuring different types of Senders, with two examples: a network packet sender and an Apache Kafka sender.

The first one, the NetworkSender, uses the Libtins library to forward the network packet on the configured interface. It does so by creating a PacketSender object from the library API and calling the send method. This object then handles the low-level operations required for converting the data into the right format and forwarding it through a specified network interface, which is configurable by passing an input string to the object constructor.

The second type of sender deals with the communication with Apache Kafka and ksqlDB. This is carried out by using a C++ library called librdkafka, which “is a C library implementation of the Apache Kafka protocol, providing Producer, Consumer and Admin clients” [18]. This library has been chosen because it provides both message delivery reliability and high performance, being able to send more than one million messages per second for the producer, which, in our case, means that we could theoretically support sending up to one million network packets per second from our sniffer to the topic.

Leveraging the power of this library, we have created a wrapper class, called KafkaSender, which is responsible for creating a librdkafka producer that will send messages to Apache Kafka. Objects of this type can be constructed by providing a broker and topic name. This will ensure that the messages will be sent to the right place and stored in the database at the appropriate location. Furthermore, during the construction of the object, a Kafka producer is dynamically spawned with the responsibility of propagating the input messages to the database. Additionally, the user will be notified about the success or failure of the sending operation by the delivery report member variable.

By using a network packet queue, we ensure that each popped message is processed and passed to the sender immediately and afterwards delivered to the Apache Kafka topic, which essentially makes the communication between our application and Apache Kafka real-time, as the processing step only involves converting our network packet to JSON, which, while not being negligible, is not a performance intensive task.

Considering all the above, the application strongly aims to provide the smallest time possible between the capture of the data and its storage in the topic, which is a big advantage when talking about the speed of processing and querying the network packets inside Kafka using KSQL queries. We can consider this a vast improvement when comparing it to our previous approach detailed in [14], where all the captured messages were written on a disk, and later ingested into Kafka in bulk.

## 3. Results

This section presents details on setting up the testing environment and the process of ingesting network packets into an Apache Kafka topic. It also details how to visualize the network packets from the initial capture up to when they are stored inside the topic.

### 3.1. Setting up the Test Environment

The test environment is similar to the system presented in Figure 1. This consists of one to multiple workstations that are connected to the Local Area Network and sending/receiving internet traffic. The Netlog software is executed on one of the devices connected to the monitored network, captures relevant traffic, and transmits the extracted customized information for ingestion into a Kafka topic.

In order to setup real-time communication with Apache Kafka, which is one of the main points we wish to illustrate in this research paper, we will need to first start the Confluent platform, consisting of Apache Kafka and ksqlDB, along with the ksql CLI, as explained in [18,19], and then run our custom packet capture tool to publish the network packets to the Kafka topic.

Running the Confluent platform can be easily conducted by cloning our fork available in [20] of the ids-ksql project [21] and executing the following command inside the root directory: docker-compose up -d. This command uses docker compose, which is a “tool for defining and running multi-container Docker applications” [22].

By running the command described above, the previously described container stack will be started, containing Apache Kafka and ksqlDB. After the two docker containers are all set up, we can proceed by creating a topic inside of which our captured packets will reside. This can be performed by executing the following command: *docker-compose exec kafka kafka-topics –zookeeper zookeeper:2181 –create –topic network-traffic –partitions 1 –replication-factor 1*, as explained in [18].

Once the topic for the network traffic has been created, the next step in setting up our example involves connecting to ksqlDB. This is required to visualize our data as it enters the Kafka topic through the conveniently provided interface. To start the command line interface for ksqlDB, called ksql CLI, the following command must be run: *docker-compose exec ksql-cli ksql http://ksql-server:8088*. This starts the command line interface listening to the ksql server at port 8088. After executing this command, we should be presented with the interactive ksql console, from which we can execute operations on the database.

With the connection being established and the CLI for ksqlDB running, we first need to make sure that our network traffic topic was created. This can be carried out by running the show topics command. Afterwards, peeking into the topic to visualize the incoming network traffic is as easy as running the command: print ‘network-traffic’. Granted everything was performed properly and we encountered no errors, we should be able to see our newly created empty topic displayed on the interface.

All the above having been carried out, we can begin capturing packets and sending network traffic to Apache Kafka in real-time by running our application with administrative privileges and the following command-line arguments: *netlog -i <network_interface> --live –sender kafka –broker localhost:19092 –topic network-traffic*. This command starts a new traffic capture using our tool on interface network-interface and instructs the output to be sent to Apache Kafka to the specified broker and topic name.

After executing the command, we should begin seeing network packets converted into JSON format flooding the Kafka topic we are visualizing inside the ksql console. From this point on, we can use the powerful queries provided by ksqlDB to match the traffic against certain patterns according to the specific use-case. An example of this has been detailed in [14], where we used these queries to match against suspicious activity for the purpose of intrusion detection.

### 3.2. Real-Time Communication with Apache Kafka ksqlDB

**Figure 7:**
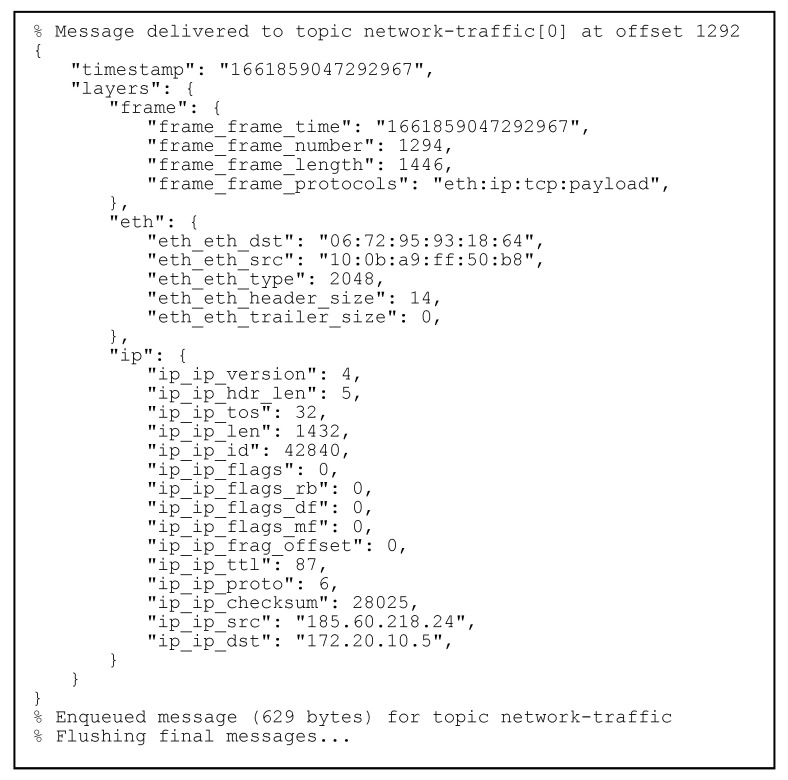
Sending messages.

In Figure 7, we can observe the command-line output of the network logging tool, for one of the captured packets.

In the message above, the tool reports delivering a message to the network-traffic topic which we created previously at a specific offset. The contents of this message can be seen as JSON, and we can identify that we have encountered a TCP network packet, with basic contents such as those of the Ethernet and IP protocol processed in more detail by our tool.

**Figure 8:**
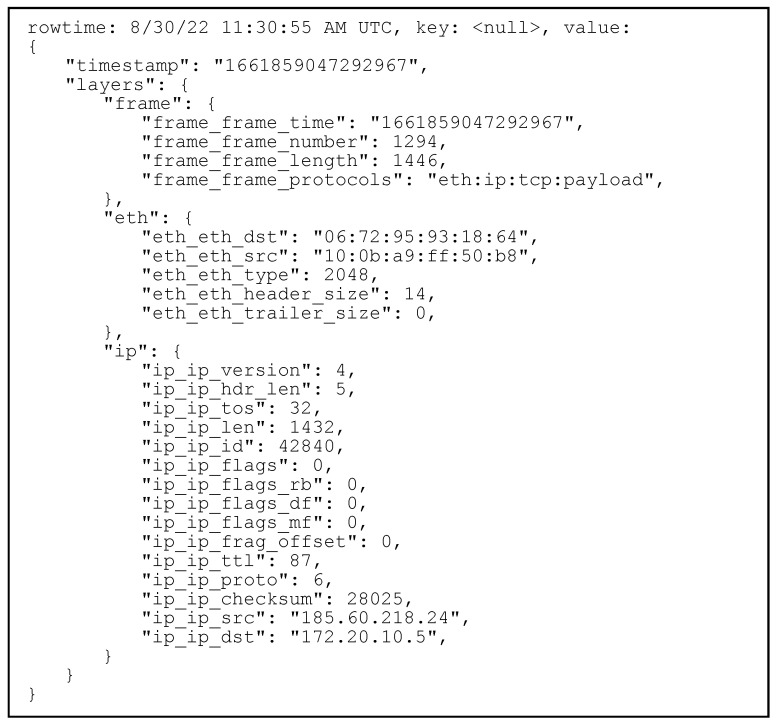
Message ingested in a Kafka topic.

To verify this received packet, we can have a look on the other side, in the ksql CLI, where we can see that the message was successfully received and stored in the topic. Furthermore, we can see the fact that the two packets are indeed identical, proving that the communication between the two systems works as intended. This is illustrated in Figure 8.

### 3.3. Performance Comparison to the Previous Solution

The main performance considerations in our use-case can be summed up to how fast our application can produce a network packet to be ingested into Apache Kafka. This can be envisioned as the time it takes from the actual interception of the packet to the moment it is available for querying in Apache Kafka. In the following paragraphs, we shall see how improvements have been made to a previous solution we developed, to facilitate a faster packet ingestion time. We shall also look at how the overhead introduced by processing packets sequentially was a major factor in the previous solution, and reason about how irrespective of the number of input packets, parallelizing the packet processing and forwarding approach yields the biggest performance gain in the case described above.

Capturing network packets using the solution discussed in this research paper utilizes multi-threading to boost performance. When analyzing the performance gain, one is likely to first consider the packet processing time and base their comparison off that alone. As we shall see, however, that does not provide the whole picture, missing a few important details.

**Figure 9:**
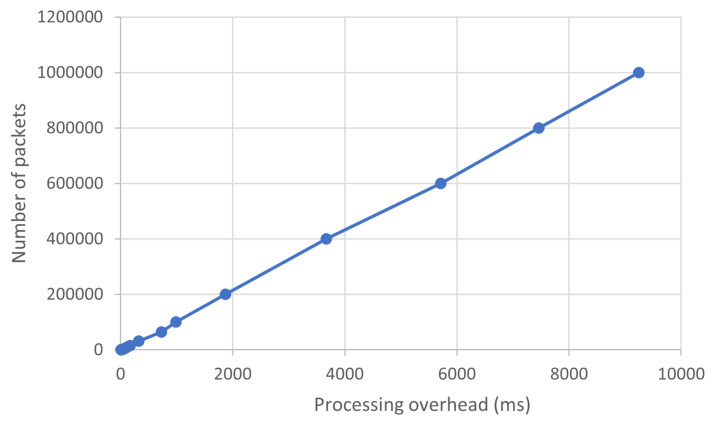
Overhead measurement for the previous solution presented in [14].

The previous work conducted on this, as seen in [15], followed a sequential approach, meaning that our tool first had to capture all the desired network packets and store them locally, on a disk, before another component of the system could start the ingestion process by reading the stored files and then forwarding the data to Apache Kafka. As a result, the overall performance was greatly impacted by this packet storage overhead, that varies mostly in a linear fashion to the number of processed packets. This can be seen in Figure 9, where the overhead was measured and charted by running the tool on different sample input sizes. We can observe that the overhead can become quite significant, reaching upwards of 9 s when processing a very large number of packets, all of this before Apache Kafka can start reading the data. In broad terms, this means that the user experiences a varied amount of delay between the packet capture and the moment they can start running queries on the traffic, which is based on the number of captured packets and grows over time. Consequently, this limitation significantly impacts the number of use-cases that can be addressed, primarily due to the inability of the solution presented in [14] to process network traffic in real-time.

After taking all of that into account, we can begin discussing the performance improvements of the current solution. Which, as opposed to the previous one, uses a multi-threaded approach, as detailed in the previous sections, and aims to improve the speed of the system by making the captured packets available for processing in Apache Kafka sooner, rather than having to wait for the sniffing process to end to start Kafka ingestion.

A direct performance comparison between the previous solution and the current one could not be charted, as doing so is not as simple as running the two tools against similar input samples, but rather requires tracing the network packets and measuring the time it takes from their actual capture to them being available inside Kafka. That being said, if we do consider the actual packet processing time as being constant, as mentioned in the introductory paragraph of this section, and are interested only in the time taken from capturing the packet to it being ingested in Kafka. We can undoubtedly conclude that splitting these tasks into separate threads of execution and forwarding the packets as soon as they are available after capture, without using intermediate storage, will yield a big performance boost, as the packets will be available in the Kafka topic far sooner than if we had to wait for the end of the capture before we could start ingesting. This means that the user-defined queries inside the database are more effective, as data are available sooner, and the final application, for example, an intrusion detection system, performs better.

## 4. Conclusions

This research paper proposed a real-time system for customized network traffic capture. The system we created encompassed the development of a software tool called Netlog that utilizes the Libtins library to extract relevant information from network traffic and send it in real-time to be ingested in Apache Kafka topics. The results obtained from the experiments further confirm the practicality of using Libtins for constructing network traffic sniffers that find applications in diverse domains. Based on the results presented illustrating that our packet capture tool can be integrated with Apache Kafka, successfully sending and receiving messages that contain customized information extracted from within the inspected network, a further case can be made for the practicality of the Libtins library in regard to building traffic analysis tools which can be used in a variety of domains that require a real-time response when the state of the system changes.

Future research could focus on integrating this tool into an actual technology stack, aiming to explore its potential in practical use-cases, such as intrusion or threat detection, general network monitoring, and more. By incorporating the tool into real-world scenarios, researchers and practitioners can delve deeper into its capabilities and advance the field of network traffic analysis and management.

## Data Availability

The data that support the findings of this study are available from the corresponding author, D.Z., upon reasonable request.
